# Microglial Mitochondrial Dysfunction: The Storm Center of Post‐Stroke Neuroinflammation

**DOI:** 10.1002/cns.71060

**Published:** 2026-07-27

**Authors:** Ruchong Fan, Chuan Wang, Zi Lin, Shiling Chen, Chao Pan, Hao Nie, Chuan Qin, Xuan Wu, Zhouping Tang

**Affiliations:** ^1^ Department of Neurology, Tongji Hospital, Tongji Medical College Huazhong University of Science and Technology Wuhan Hubei China; ^2^ Department of Geriatrics, Tongji Hospital, Tongji Medical College Huazhong University of Science and Technology Wuhan Hubei China; ^3^ Department of Neurology, Tongji Hospital, Tongji Medical College, State Key Laboratory for Diagnosis and Treatment of Severe Zoonotic Infectious Diseases Huazhong University of Science and Technology Wuhan Hubei China; ^4^ Hubei Key Laboratory of Neural Injury and Functional Reconstruction Huazhong University of Science and Technology Wuhan Hubei China; ^5^ Hubei Provincial Clinical Research Center for Stroke Wuhan Hubei China

**Keywords:** fusion/fission dynamics, metabolic reprogramming, microglia, mitochondrial dysfunction, mitochondrial transplantation, mitophagy, neuroinflammation, stroke

## Abstract

**Background:**

Stroke remains a major global cause of death and disability, with many patients either missing the therapeutic window or responding poorly to current first‐line treatments. Consequently, secondary neurological injury, driven predominantly by neuroinflammation, has emerged as a critical therapeutic target. Microglia rapidly sense post‐stroke microenvironmental changes and adopt distinct inflammatory phenotypes that shape pathophysiological outcomes.

**Results:**

Accumulating evidence, including high‐resolution spatial profiling and single‐cell omics, positions mitochondrial dysfunction at the core of these responses. This review synthesizes recent findings on microglial mitochondrial dysfunction in stroke, introducing the concept of a microglial mitochondrial “storm center”. In this model, reactive oxygen species (ROS) trigger an inflammatory cascade, while impairments in mitochondrial quality control (MQC) exacerbate pathogenic signaling. Metabolic reprogramming further sustains inflammatory polarization, influencing interactions with neurons, astrocytes, and endothelial cells.

**Conclusions:**

This “storm center” provides a conceptual framework for developing strategies to mitigate secondary brain injury. Finally, this review highlights key molecular mechanisms, potential therapeutic targets, and translational opportunities, providing a stronger foundation for future stroke research and therapeutic innovation.

## Graphical abstract

Stroke disrupts microglial mitochondrial homeostasis, leading to acute damage and metabolic dysfunction that amplify pro‐inflammatory signaling. Although quality control mechanisms initiate protective responses, persistent stress overwhelms them. The resulting imbalance drives a self‐reinforcing neuroinflammatory storm that exacerbates secondary brain injury.

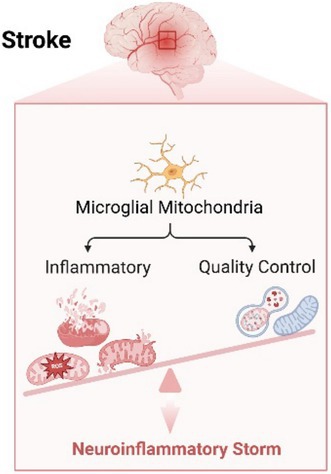

## 1 Introduction

Stroke is an acute cerebrovascular disorder and ranks as the second leading cause of death worldwide. Its global burden remains substantial and is projected to increase in the coming decades [1, 2]. Stroke can be simply divided into two major types: ischemic stroke (IS) and hemorrhagic stroke (HS) [3]. IS is typically caused by arterial occlusion, which leads to cerebral hypoxia and triggers a cascade of complex pathophysiological events [4, 5]. In contrast, HS is caused by the rupture of cerebral blood vessels, leading to brain hematoma, direct tissue damage, and secondary inflammatory responses [6, 7]. HS similarly elicits pronounced oxidative stress, neuroinflammation, and metabolic dysregulation, culminating in both primary and secondary brain injury [8, 9].

Currently, the first‐line treatment option for IS aims to restore blood flow within a time window and rescue neurons in the ischemic penumbra [10]. Minimally invasive therapies for HS are also being increasingly studied; however, a significant proportion of patients suffering from stroke remain ineligible for first‐line treatment or achieve suboptimal outcomes [11]. Over several decades, extensive studies have attempted to achieve direct neuronal protection; however, most therapeutic agents could not effectively improve patients' prognosis [12, 13]. Thus, targeting secondary neurological damage following stroke has emerged as a persistent research focus and a promising therapeutic approach.

**FIGURE 1:**
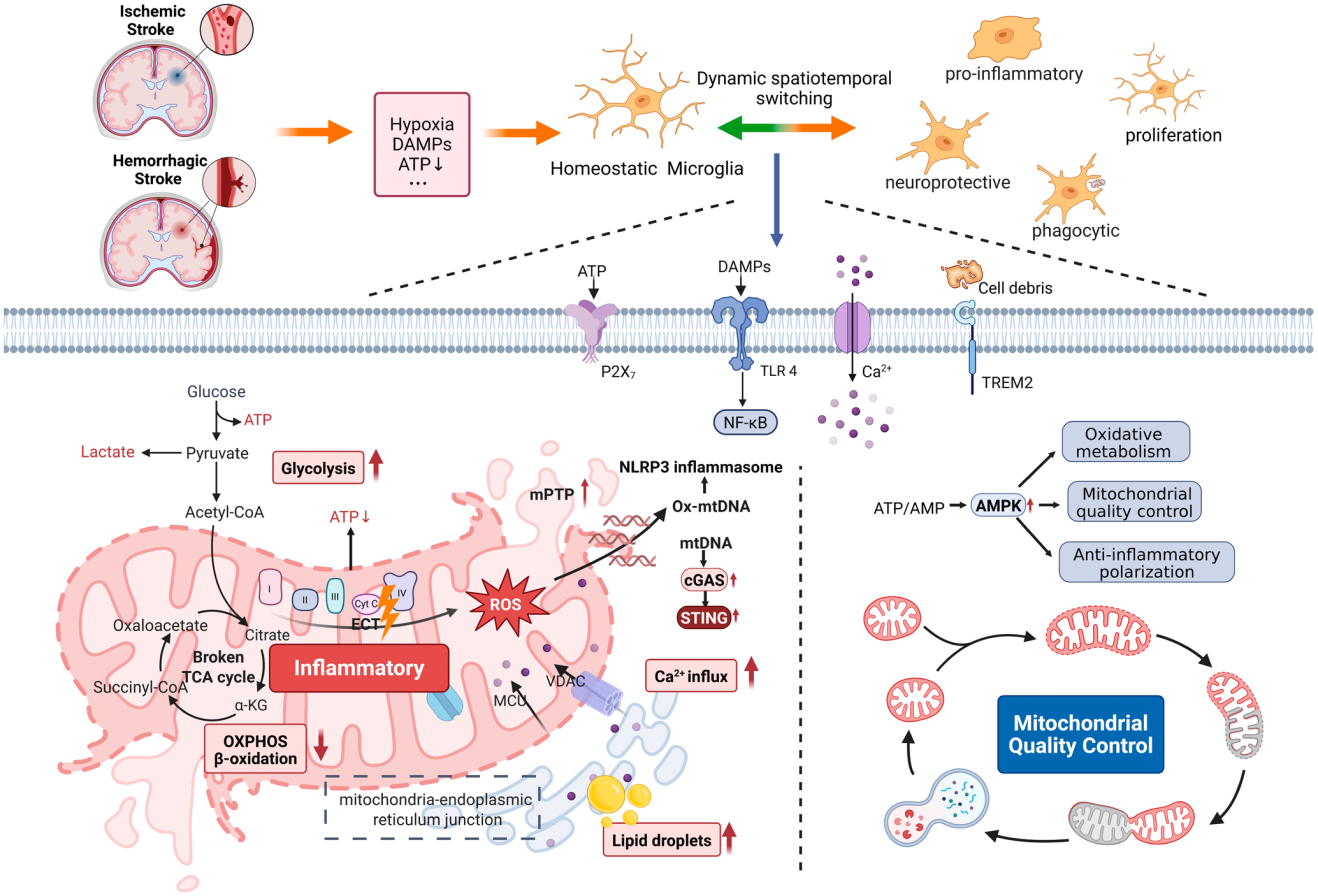
Mitochondrial inflammatory injury and protective programs in microglia after stroke. Ischemic or hemorrhagic stroke induces DAMP release, hypoxia, and rapid ATP depletion, triggering rapid microglial activation and phenotypic remodeling. Mitochondrial dysfunction promotes excessive ROS generation, Ca^2+^ overload, mPTP opening, and mtDNA leakage, which activate the cGAS–STING pathway and the NLRP3 inflammasome, together with HIF‐1α–driven pro‐inflammatory metabolic reprogramming. In parallel, AMPK‐dependent energy sensing and mitochondrial quality control eliminate damaged mitochondria and reduce oxidative stress. The balance between these opposing pathways determines microglial functional outcomes and post‐stroke neuroinflammation. Created with BioRender.com.

Accumulating evidence indicates that neuroinflammation, characterized by microglial activation and polarization, represents a critical process in exacerbating secondary damage after IS and HS [14, 15]. Following stroke onset, microglia, the resident immune cells in the brain, rapidly respond to microenvironmental changes and transition into complex, diverse functional states, including pro‐inflammatory, phagocytic, neuroprotective, and proliferation states (Figure 1) [16, 17]. The functional bias of the ultimate microglial response is highly dynamic, governed by the spatiotemporal signature of the post‐stroke environment and organism‐specific determinants.

Mitochondria, as central regulators of cellular energy and metabolism, integrate and transmit signals that orchestrate adaptive responses in microglia [18]; however, persistent mitochondrial dysfunction can lead to the entrapment of microglia in a maladaptive pro‐inflammatory state [19, 20]. Although mitochondrial structure is largely conserved across cell types, their functions largely rely on their microenvironment. In microglia, mitochondria serve not only as energy producers but also as key regulators of innate immune signaling and metabolic adaptation, thereby controlling their activation states. Mitochondrial dysfunction enhances inflammation, reduces phagocytosis, and delays tissue repair. Because of this tight immune‐metabolic coupling, microglial mitochondria are vulnerable to ischemic, reperfusion, and hemorrhagic stresses, making them active drivers, rather than passive targets, of post‐stroke neuroinflammation.

Unlike neurodegenerative disorders, stroke represents an acute stress, characterized by massive and rapid release of damage‐associated molecular patterns (DAMP), which leads to abrupt mitochondrial dysfunction within a narrow temporal window [21]. Although previous reviews have investigated the role of mitochondrial dysfunction or microglial activation independently [22, 23], the intersection of mitochondrial dysfunction and microglial activation in the context of acute stroke remains insufficiently explored. Moreover, the high evolutionary conservation of bilaterian mitochondrial genomes implies functional interchangeability of core mitochondrial signaling pathways across vertebrates, supporting the translational robustness of mitochondrial‐targeted therapies [24].

This review develops the concept of microglial mitochondrial “storm center” and discusses post‐stroke neuroinflammation along disease progression. Within this framework, mitochondrial dysfunction is viewed not merely as a downstream consequence of injury but as a key regulator of microglial functional states and inflammatory outcomes following stroke. Building upon recent advances in mitochondrial biology and microglial heterogeneity, this review summarizes emerging mechanisms underlying post‐stroke neuroinflammation and highlights mitochondria‐centered therapeutic opportunities to inform future translational research.

## 2 Literature Search Strategy

A comprehensive literature search was conducted in the PubMed database using the keywords “microglia”, “mitochondrial dysfunction”, “stroke”, “ischemic stroke”, “intracerebral hemorrhage”, “neuroinflammation”, and “energy metabolism”. Considering the rapid advances in mitochondria‐targeted therapies and their ongoing clinical translation, a comprehensive review focusing on mitochondrial function in microglia is both timely and essential.

## 3 Microglial Mitochondrial Structure and Dysfunction

Mitochondria are highly dynamic, compartmentalized organelles whose double‐membrane structure can help regulate oxidative stress, organelle renewal, signal transduction, and cell survival [25, 26]. The outer mitochondrial membrane (OMM) contains voltage‐dependent anion channels (VDAC) for exchanging metabolites. The inner mitochondrial membrane (IMM) is folded into cristae that house the electron transport chain (ETC) complexes and ATP synthase, which drive oxidative phosphorylation (OXPHOS) and maintain the mitochondrial membrane potential (MMP, ΔΨm). Pro‐apoptotic factors, such as cytochrome C, are sequestered in the intermembrane space (IMS) and released subsequent to membrane permeabilization, thereby triggering apoptosis and inflammatory signaling. The mitochondrial matrix contains enzymes that catalyze the tricarboxylic acid (TCA) cycle, fatty acid β‐oxidation, and mitochondrial DNA (mtDNA), supporting both metabolic and genetic functions [27]. This compartmentalization also makes mitochondria highly vulnerable to oxidative stress and ΔΨm collapse.

Although this structural framework is conserved across different cell types, the functional deployment of mitochondria highly relies on the context. In microglia, mitochondria not only serve as bioenergetic organelles but also are tightly linked to innate immune signaling and metabolic adaptation, critically regulating microglial activation states rather than merely controlling cell survival. In neurons, mitochondrial dysfunction results in bioenergetic failure and cell death, whereas in astrocytes, mitochondrial dysfunction disrupts metabolic support and homeostatic regulation. In contrast, mitochondrial dysregulation in microglia preferentially enhances inflammatory signaling, impairs phagocytic function, and delays tissue repair. This immune‐metabolic coupling renders microglial mitochondria particularly sensitive to ischemic stress, reperfusion injury, and hemorrhagic stresses, imposing exceptional demands on MQC and metabolic reprogramming. Accordingly, microglial mitochondria actively regulate post‐stroke neuroinflammatory dynamics rather than being passive targets of injury.

## 4 Spatiotemporal Heterogeneity of Microglial Mitochondrial Responses After Stroke

**FIGURE 2:**
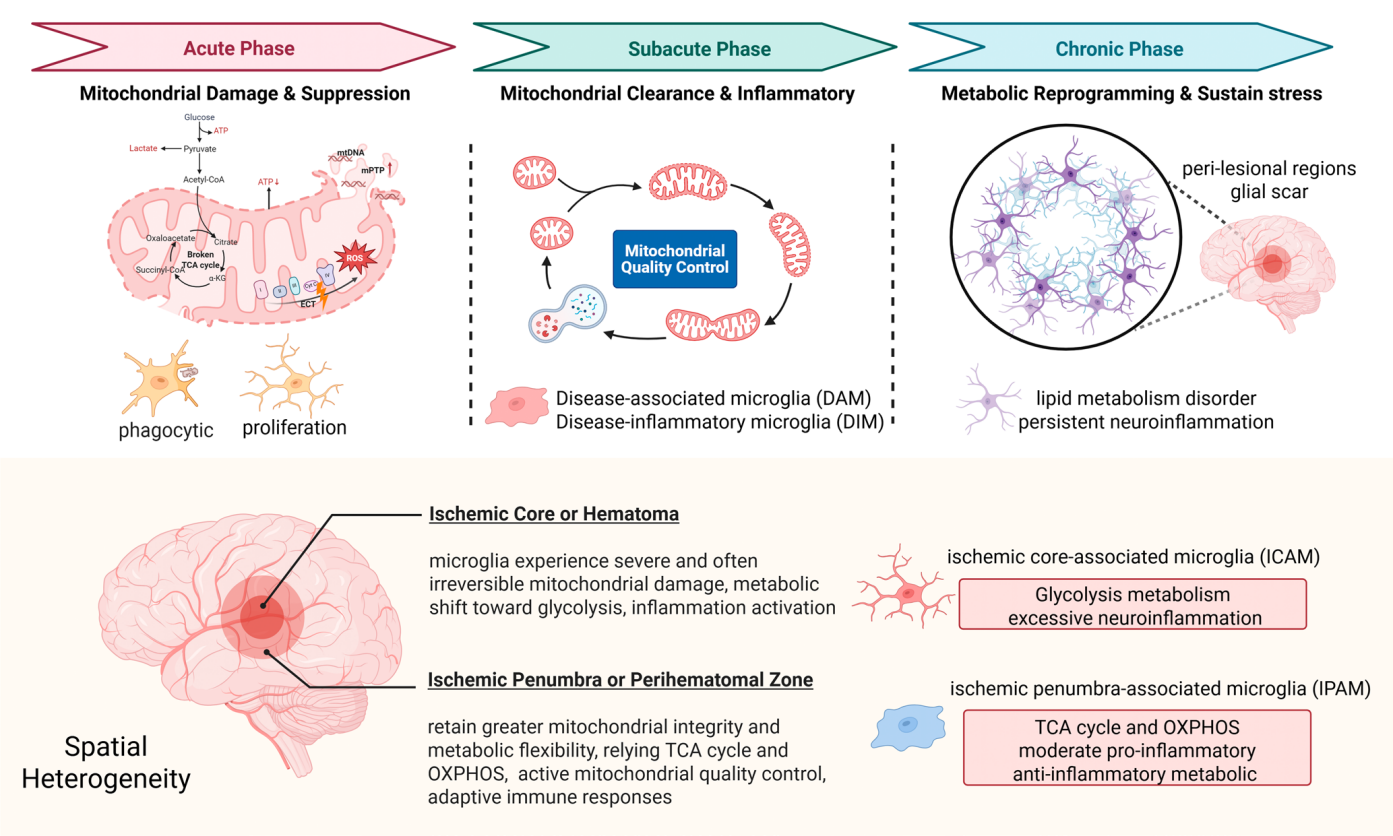
Spatiotemporal dynamics of microglial mitochondrial remodeling after stroke. Single‐cell and spatial transcriptomics reveal that microglial mitochondrial responses evolve across both time and space after stroke. During the acute phase, electron transport chain dysfunction and excessive mitochondrial ROS drive glycolytic switching, proliferation, and phagocytic activation. In the subacute phase, mitochondrial quality control programs are upregulated, with microglia adopting inflammatory states (DAM/DIM). In the chronic phase, persistent mitochondrial stress promotes lipid metabolic disorder, sustained neuroinflammation, and glial scar formation. Spatially, microglia in the stroke core exhibit severe mitochondrial damage and strong inflammatory activation, whereas peri‐lesional microglia retain greater mitochondrial plasticity. Distinct subsets include pro‐inflammatory ICAM and metabolically adaptive IPAM. Created with BioRender.com.

Recent advances in single‐cell RNA sequencing (scRNA‐seq) and spatial transcriptomic (ST) technologies have revealed that microglial responses to stroke are highly heterogeneous, both temporally and spatially (Figure 2). Importantly, mitochondrial alterations in microglia evolve dynamically in response to local microenvironmental cues.

From a temporal perspective, post‐stroke microglial mitochondrial dysfunction evolves through distinct and highly dynamic functional stages. In the acute phase, abrupt cerebral ischemia or hemorrhage results in rapid ATP depletion, mitochondrial membrane depolarization, and early disruption of redox homeostasis. These alterations are primarily driven by damage to the ETC and overproduction of ROS [27], which act as DAMPs and activate microglia [28, 29]. Concomitantly, microglia exhibit pronounced proliferative response and enhanced phagocytic capacity [30].

In the subacute phase, mitochondrial quality control (MQC) mechanisms, including mitophagy, mitochondrial biogenesis, and fusion‐fission dynamics, are robustly engaged to restore mitochondrial integrity and metabolic function [31]. At this stage, microglia primarily exhibit an inflammatory phenotype, referred to as disease‐associated microglia (DAM) and disease‐inflammatory microglia (DIM) [30].

In the chronic phase, persistently activated microglia recruit and activate astrocytes, leading to glial scar formation in peri‐lesional regions [32, 33]. Sustained mitochondrial stress and long‐term metabolic reprogramming perpetuate neuroinflammation or impair reparative processes, thereby exacerbating secondary brain injury and hindering functional recovery.

Spatially, the severity of regional injury significantly affects the mitochondrial response of microglia [34]. In the stroke core, microglia experience severe and often irreversible mitochondrial damage, accompanied by marked proliferative responses. Microglia located in peri‐infarct regions, such as the ischemic penumbra in IS and the perihematomal zone in HS, retain greater mitochondrial plasticity, which allows for more adaptive functional responses.

Li et al. employed scRNA‐seq and ST technology to classify microglial populations based on their spatial localization in the mouse models of middle cerebral artery occlusion (MCAO) during the hyperacute and acute phases of stroke [35]. They identified ischemic core‐associated microglia (ICAM) and ischemic penumbra‐associated microglia (IPAM). The study observed a gradient distribution of immune activation. Specifically, the intensity of inflammation progressively diminished with increasing distance from the infarct core. ICAM microglia primarily relied on glycolytic metabolism, leading to excessive neuroinflammation and more severe tissue injury. In contrast, IPAM microglia predominantly adopted the TCA cycle and oxidative phosphorylation, exhibiting moderate pro‐inflammatory activity and anti‐inflammatory metabolic characteristics.

In intracerebral hemorrhage (ICH), perihematomal edema (PHE) manifests as increased water content in parenchymal tissue adjacent to the hematoma [36]. Its progression is widely regarded as a quantifiable marker of SBI [37]. The first scRNA‐seq analysis of human PHE tissue systematically delineated the immune landscape, revealing that most microglia exhibit a pronounced pro‐inflammatory phenotype within 48 h after bleeding [38]. These findings further confirm the central role of microglia‐driven inflammation in the pathogenesis of SBI after ICH. However, current research on mitochondrial dysfunction in ICH remains limited and warrants further studies.

Collectively, evidence from both temporal and spatial dimensions redefines the role of post‐stroke mitochondrial dysfunction in microglia as a heterogeneous, context‐dependent, and dynamically regulated process, rather than a uniform downstream consequence of injury. This conceptual framework highlights the need for integrating temporal progression with spatial localization when interpreting mitochondrial pathology in microglia and provides a theoretical basis for developing stage‐specific and region‐specific therapeutic strategies targeting microglial mitochondria to modulate post‐stroke neuroinflammation.

## 5 Early Mitochondrial ROS Burst Triggers Microglial Inflammatory Activation

### 5.1 Mitochondrial ROS Accumulation and Redox Imbalance

**FIGURE 3:**
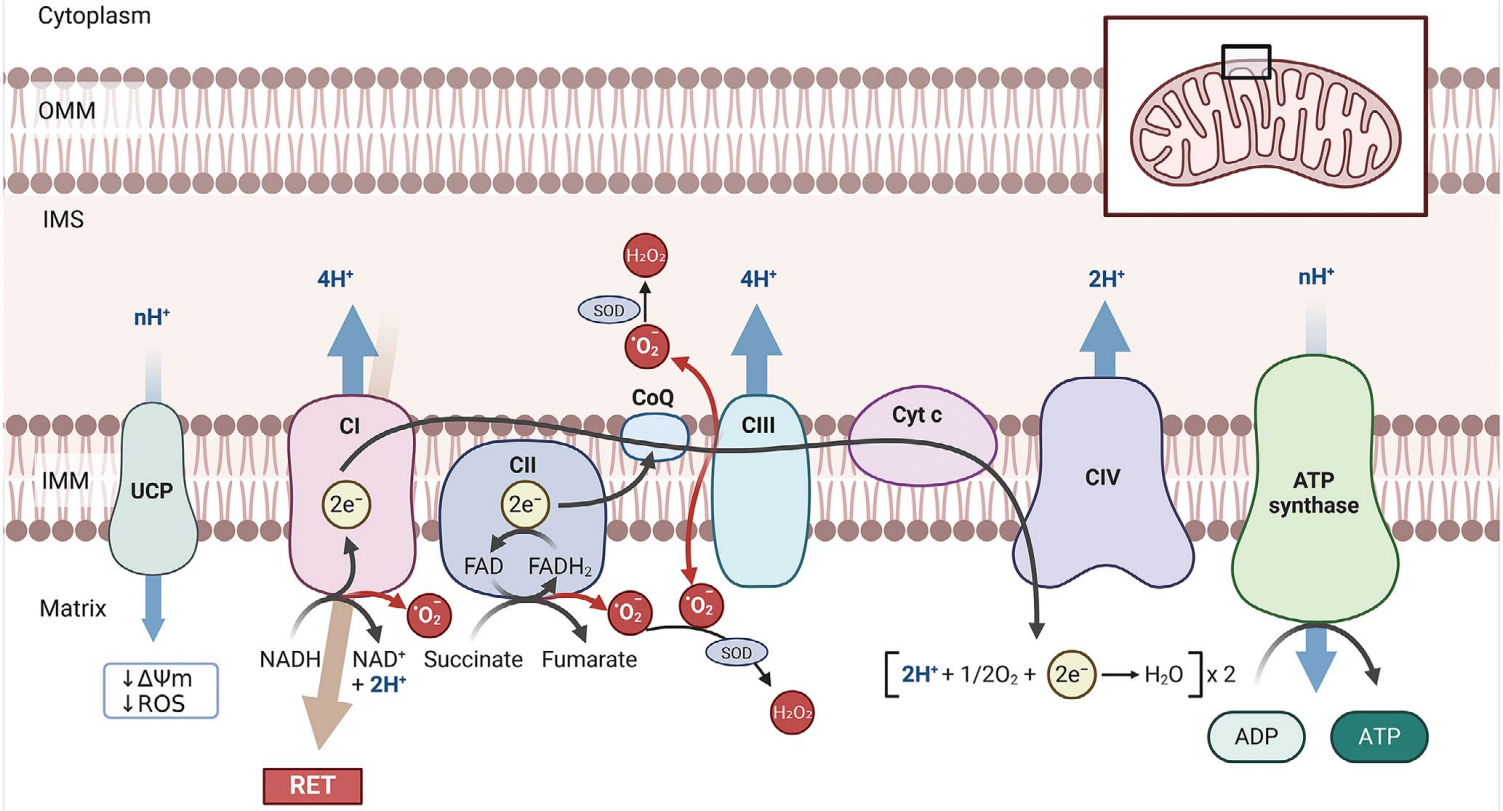
Mitochondrial ROS generation and regulation in the electron transport chain. Potential sites of mitochondrial ROS generation within the electron transport chain (ETC). Electron leakage at complexes I–III produces superoxide (O_2_^−^), which can be converted to hydrogen peroxide (H_2_O_2_). Notably, ETC dysfunction drives reverse electron transport (RET) at Complex I, making it a potent source of excessive superoxide burst. As a key regulatory mechanism, uncoupling proteins (UCPs) facilitate a mild proton leak across the inner mitochondrial membrane, reducing ΔΨm and limiting excessive ROS production, thereby providing mitochondrial protection. Created with BioRender.com.

During the early acute phase after stroke, mitochondrial dysfunction rapidly induces excessive ROS production in microglia, which acts as a primary trigger for inflammatory activation and secondary brain injury [39, 40, 41]. Under physiological conditions, the ETC generates ATP through OXPHOS (Figure 3). Under pathological conditions, ETC impairment, particularly at complexes I and III, disrupts the forward flow of electrons to complex IV, forming a high ΔΨm that facilitates reverse electron transport (RET) at complex I, a potent source of superoxide generation [42]. Superoxide dismutases (SOD) rapidly convert the resulting superoxide (O_2_•^−^) ions to hydrogen peroxide (H_2_O_2_) [43]. Excessive ROS production amplifies ROS‐induced ROS release (RIRR), forming a self‐perpetuating oxidative cycle throughout the mitochondrial network [44]. ROS accumulation leads to widespread oxidative damage to lipids, proteins, and nucleic acids, compromising membrane integrity, enzymatic activity, and genomic stability, ultimately resulting in apoptotic or necrotic death [45].

Ischemic and hemorrhagic stroke impose distinct oxidative stress paradigms: ischemia primarily induces hypoxia‐driven electron transport chain dysfunction and reperfusion triggers ROS overproduction, whereas hemorrhage is primarily driven by hemoglobin degradation products and iron overload, which generate highly reactive hydroxyl radicals (•OH) through the Fenton reaction, thereby amplifying oxidative damage.

Mitochondrial uncoupling proteins (UCPs) are proton transporters located in the IMS. Unlike the mitochondrial toxicity of classical uncouplers, UCPs mediate mild proton leakage that lowers ΔΨm, prevents ROS bursts, and maintains redox homeostasis [46]. Among them, UCP2 is highly expressed in neurons and microglia [47], which was shown to downregulate IL‐1β and TNF‐α, upregulate IL‐10 and TGF‐β via the AMPKα/NRF1 pathway, and protect against ferroptosis in experimental stroke [48].

### 5.2 Ox‐mtDNA‐Driven Inflammasome Activation

Post‐stroke overproduction of ROS and DAMPs provokes mitochondrial permeability transition pore (mPTP) opening and oligomerization of VDACs, thereby facilitating the release of mtDNA into the cytoplasm [49, 50, 51]. As a circular, double‐stranded molecule, mtDNA acts as a DAMP that induces inflammatory signaling [50, 52]. In microglia, ROS and oxidized mitochondrial DNA (ox‐mtDNA) generated during mitochondrial dysfunction together activate the NLRP3 inflammasome, thereby driving caspase‐1‐dependent IL‐1β maturation and pro‐inflammatory microglial responses [53]. Therefore, limiting the production of mtROS is essential for inhibiting stroke‐associated mitochondrial injury.

The cGAS/STING pathway is a major mtDNA sensor involved in post‐stroke inflammation. Cytosolic mtDNA activates cyclic GMP‐AMP synthase (cGAS), generating cyclic GMP‐AMP, which in turn activates STING, as the key mediator of the interferon response [54, 55, 56]. rFGF21 blocked mtDNA release into the cytoplasm and inhibited the cGAS/STING signaling pathway [57].

The inflammasome, a multiprotein complex, is a key driver of post‐stroke inflammation [58]. Mitochondrial dysfunction plays a pivotal role in the activation of the NLRP3 inflammasome during IS or ICH [58, 59, 60]. The well‐known mitochondrial protective agent idebenone was shown to inhibit the binding of Ox‐mtDNA to NLRP3, thereby alleviating neurological dysfunction in ischemia–reperfusion (I/R) models, defined as transient cerebral artery occlusion followed by reperfusion [61]. AIM2 is another type of inflammasome. Elevated levels of mtDNA in perihematoma microglia activate the AIM2 inflammasome. The AIM2 antagonist P202 or mitochondrial division inhibitor Mdivi‐1 attenuated neuroinflammation and improved outcomes after ICH [62].

## 6 Bioenergetics and Metabolic Reprogramming of Mitochondrial Microglia

After a stroke, microglia rapidly extend processes, migrate to injury sites, and engage in phagocytosis, processes that require high amounts of ATP [63, 64]. Mitochondrial cristae are the sites of OXPHOS, whose integrity is crucial for maintaining ΔΨm and regulating neuroinflammatory responses. ST technologies identified cysteine protease activation and recruitment domain 19 (CARD19) as a major regulator of cristae integrity, representing a novel molecular target involved in post‐stroke neuroinflammation [65]. Following stroke, microglia undergo spatiotemporally stratified metabolic reprogramming that closely parallels mitochondrial dysfunction and transitions to the inflammatory state.

During the hyperacute and acute phases, hypoxia and inflammatory signaling drive rapid glial cell reprogramming toward a glycolytic metabolic mode, a process frequently associated with increased expression of key glycolytic regulators, such as pyruvate kinase M2 (PKM2), hypoxia‐inducible factor‐1α (HIF‐1α), and hexokinase 2 (HK2) [66, 67, 68]. This metabolic transition not only supports rapid ATP generation but also sustains the transcription of pro‐inflammatory genes. Pharmacological activation of PKM2 has been shown to promote reparative microglial polarization, enhance phagocytosis, and reduce infarct volume and neuroinflammation, partly by limiting the nuclear translocation of PKM2 and suppressing HIF‐1α‐dependent glycolytic signaling [68].

Stroke also alters the functional state of microglia by modulating gene expression and the accumulation of metabolic byproducts. Enhanced glycolysis increases lactate production, which functions both as a signaling molecule and as an epigenetic modifier through histone lactylation, thereby controlling the expression of genes associated with inflammation and tissue repair [69]. Histone lactylation, particularly H3K9la, enhances transcription of glycolytic and inflammatory genes, including LDH‐A and HIF‐1α, reinforcing a feed‐forward loop that sustains glycolysis and inflammatory activation [66]. MeCP2 lactylation enhances HK2 transcription and activates the HK2/mTOR pathway, leading to mitochondrial dysfunction, glycolytic dependence, and persistent inflammatory signaling [70]. Disruption of the mitochondrial TCA cycle leads to the accumulation of citrate, succinate, and itaconate, which act as intracellular and extracellular inflammatory mediators [71, 72, 73]. Succinate accumulation in ICH can activate UCP2, thereby suppressing mtROS and microglial inflammation and exerting neuroprotectiveeffects [74].

During the subacute phase of injury, particularly in periventricular areas, some microglia initiate MQC processes. They progressively restore oxidative metabolism through the synergistic activation of OXPHOS and fatty acid β‐oxidation (FAO), thereby supporting phagocytosis and tissue remodeling [75, 76]. FAO is closely linked to lipid droplet dynamics, with liposomes originating from the endoplasmic reticulum to supply substrates for β‐oxidation. As plastic organelles, lipid droplets (LDs) accumulate extensively after stroke, particularly in astrocytes and microglia [77]. Moderate lipid droplet formation helps buffer lipid peroxidation and lipotoxicity, whereas excessive lipid accumulation is associated with metabolic disorders and pro‐inflammatory phenotypes [78, 79]. Microglia‐specific FABP4 upregulation in ICH is associated with poor neurological outcomes, whereas Fabp4 deficiency mitigates lipid droplet accumulation and improves tissue and functional recovery [80]. Studies have shown that reducing ischemia‐induced LD accumulation in microglia can upregulate carnitine palmitoyltransferase 1A (CPT1A), the rate‐limiting enzyme involved in mitochondrial β‐oxidation, enhance the synthesis of glycerophospholipids for membrane stability, suppress inflammation, and improve phagocytosis [81]. Furthermore, the immune‐metabolic receptor TREM2 on the surface of microglia enhances the expression of lipid‐sensing, metabolic, and phagocytic genes, thereby promoting liposome clearance and facilitating neuroprotective phenotypic conversion after stroke [82, 83].

In the chronic phase, specific subpopulations of microglia progressively accumulate lipids and cholesterol, accompanied by diminished clearance capacity and persistent neuroinflammation [84]. Metabolic reprogramming of cholesterol is considered a key driver of chronic neuroinflammation. The transcription regulator ATF3 may link altered lipid metabolism to pro‐inflammatory activation by regulating lipid remodeling pathways, such as CH25H and SPP1 [75]. Conversely, enhanced cholesterol efflux via CYP46A1 activation can alleviate lipid overload and facilitate white matter repair [84].

Dyslipidemia is particularly lethal in hemorrhagic strokes, where lipid peroxidation constitutes a primary source of secondary injury [85]. Excessive iron ion release from blood degradation products further exacerbates oxidative damage to lipid molecules, leading to the accumulation of ROS and toxic aldehydes, such as 4‐hydroxy‐2‐nonenal and malondialdehyde. These oxidized lipid derivatives trigger glial cell activation, neuronal apoptosis, and enhanced inflammatory signaling. In ICH, erythrocytes and their lysates drive iron accumulation in adjacent microglia, with mitochondria serving as crucial organelles for iron metabolism, utilization, and storage. Upregulation of the mitochondrial iron transporter SLC25A28 promotes aerobic glycolysis and microglial activation, while microglia‐specific deletion of SLC25A28 suppresses neuroinflammation [86].

Metabolic reprogramming of microglia is primarily driven by the synergistic regulation of energy‐sensing and oxygen‐sensing pathways, with AMP‐activated protein kinase (AMPK) serving as a central regulatory hub. As a cellular energy sensor, AMPK is activated when the ATP/AMP ratio decreases, promoting oxidative metabolism, MQC, and anti‐inflammatory polarization, suppressing HIF‐1‐dependent glycolysis and inhibiting the expression of pro‐inflammatory genes [87]. Beyond intrinsic mitochondrial mechanisms, the mitochondria‐endoplasmic reticulum contact sites (MERCS) constitute a highly specialized interface that coordinates lipid exchange and metabolic adaptation through calcium signaling [88]. After stroke, compromised MERCS integrity may exacerbate mitochondrial calcium overload in microglia, impair bioenergetic flexibility, and amplify inflammatory signaling [89, 90, 91]. Notably, the role of MERCS in the pathogenesis of stroke remains a relatively emerging research field. Direct evidence regarding their specific contribution to mitochondrial dysfunction in post‐stroke microglia is currently lacking, necessitating further validation through experimental and clinical studies.

Microglial phenotypes undergo dynamic transitions that directly affect energy homeostasis and ROS production, thereby affecting neural network functionality and modulating the integrity of the blood–brain barrier (BBB) [92, 93]. Understanding microglial metabolic‐polarization dynamics offers new therapeutic avenues for minimizing post‐stroke injury by targeting metabolic reprogramming beyond anti‐inflammatory strategies. Future studies must define the signals guiding this process toward protective functions or irreversible metabolic cell death.

## 7 Autophagy and Mitochondrial Quality Control Dysfunction in Microglia After Stroke

**FIGURE 4:**
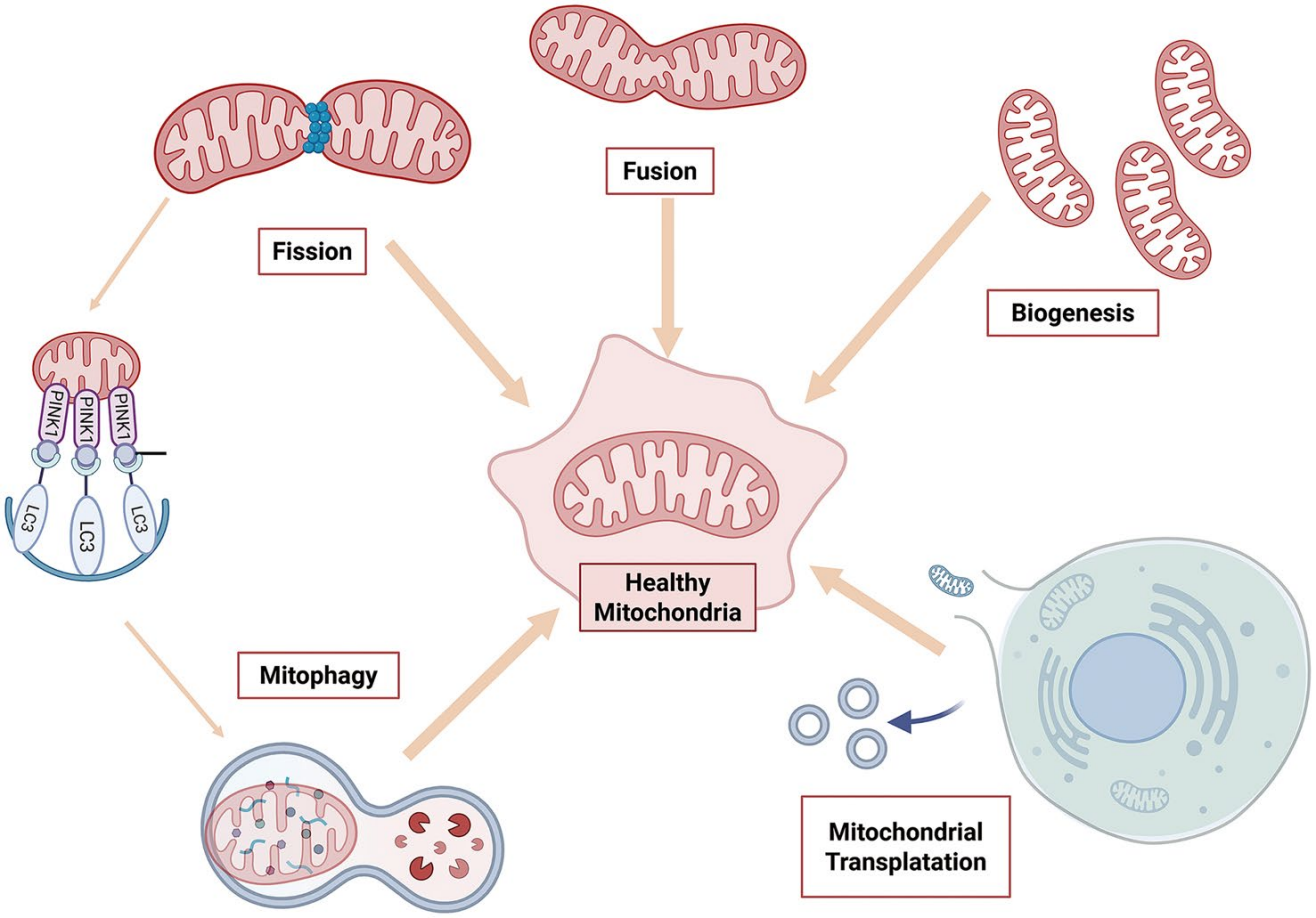
Key processes of mitochondrial quality control in microglia. Mitochondria undergo multiple dynamic processes, including mitophagy, biogenesis, fission–fusion dynamics, and intercellular mitochondrial transplantation. These mechanisms collectively maintain mitochondrial homeostasis and ensure proper cellular adaptation under stress conditions. Created with BioRender.com.

Mitochondrial dysfunction plays a central role in the pathogenesis of stroke. To cope with various cellular insults, mitochondria rely on multi‐layered quality control systems, including mitophagy, biogenesis, and fission‐fusion dynamics [94] (Figure 4). Dysregulation of these pathways exacerbates mitochondrial damage, leading to cell death and further neurological deficits. Following stroke, microglia experience profound mitochondrial stress and rely heavily on mitochondrial quality control (MQC) mechanisms to restore metabolic homeostasis and limit excessive neuroinflammation.

### 7.1 Mitochondrial Autophagy: The Clearance Pathway

Mitochondrial autophagy, also known as mitophagy, selectively removes damaged mitochondria to maintain mitochondrial quality and prevent ROS accumulation and DAMP release [95, 96]. In post‐stroke microglia, this process functions as a critical inflammatory checkpoint rather than a simple housekeeping mechanism. Ineffective removal of damaged mitochondria leads to mtROS accumulation and mtDNA leakage, thereby activating inflammasomes and reinforcing pro‐inflammatory polarization.

Among numerous genes regulating MQC, PINK1 and Parkin are the most extensively studied [97]. After mitochondrial damage, PINK1 accumulates on the OMM and activates the E3 ubiquitin ligase Parkin, which ubiquitinates OMM proteins to initiate selective mitophagy [98, 99]. Analyses of mouse models of ICH and patient samples revealed that ineffective mitophagy occurs predominantly in microglia, and PINK1 overexpression alleviates ICH‐associated brain damage [100].

Besides, peroxisome proliferator‐activated receptor gamma coactivator‐1α (PGC‐1α) was found to be upregulated in microglia after ischemic stroke [101], suggesting that PGC‐1α may be a promising therapeutic target for acute ischemic stroke. PGC‐1α serves as a key co‐regulator of gene expression in mitochondrial biogenesis and promotes autophagy and mitophagy via ULK1 [102, 103, 104]. These processes ensure the renewal of a functionally competent mitochondrial network.

The protective and detrimental effects of mitochondrial autophagy in microglia after stroke remain controversial [105, 106, 107, 108]. We propose that the functional outcome primarily depends on the dynamic balance between the extent of mitochondrial damage and the capacity for mitochondrial autophagy. Acute ischemia–reperfusion often induces rapid and intense mitochondrial stress responses, potentially leading to excessive or dysregulated autophagy and tissue injury. Conversely, hemorrhagic stroke typically leads to persistent and profound mitochondrial damage accompanied by insufficient or dysfunctional mitochondrial autophagy. In such cases, stronger mitochondrial autophagy can help clear damaged mitochondria and suppress inflammation. Therefore, maintaining microglial metabolic homeostasis relies more on moderate and efficient autophagic flux than on maximally activated autophagy.

### 7.2 Mitochondrial Biogenesis: The Renewal Pathway

Mitochondrial biogenesis is the process through which new mitochondria are generated via the proliferation of existing organelles and replication of mtDNA [109]. Newly formed mitochondria replace dysfunctional and damaged organelles, which are selectively degraded by mitochondrial autophagy. During the subacute and recovery phases after stroke, restoration of mitochondrial homeostasis is particularly critical for microglial functional reprogramming and tissue repair.

Transcription factor A (TFAM) packages and compacts naked mtDNA into nucleoid‐like structures, minimizing susceptibility to mtROS [110]. Timely termination of mtDNA‐induced inflammation promotes tissue repair. UCP2 promotes mitochondrial biogenesis in microglia via AMPKα/NRF1‐mediated upregulation of NRF1 and TFAM, supporting stroke recovery [48]. Treatment with cannabidiol (CBD) was shown to upregulate TFAM, prevent microglial activation, and improve mitochondrial defects in MCAO models [111]. Additionally, a CBD‐based drug delivery system further enhanced targeted restoration of mitochondrial function [112].

Cytidine/uridine monophosphate kinase 2 (CMPK2), a rate‐limiting enzyme involved in the synthesis of deoxyribonucleoside triphosphates (dNTP), regulates mtDNA replication [50]. CMPK2 overexpression was shown to trigger the synthesis of exposed and loosely packed mtDNA in lipopolysaccharide (LPS)‐exposed immune cells, thereby triggering inflammasome‐mediated inflammation [113].

Collectively, mitochondrial biogenesis and mtDNA handling represent a delicate balance: renewal promotes functional recovery, whereas mtDNA leakage enhances neuroinflammatory cascades. Therefore, fine‐tuning these processes is critical for achieving neuroprotection after stroke.

### 7.3 Mitochondrial Fusion and Fission Dynamics: Structural Remodeling

Mitochondrial dynamics, encompassing fusion and fission, control mitochondrial shape, number, distribution, and functional state [114]. Fusion allows content exchange between different mitochondria, repairing damaged organelles, whereas fission facilitates mitochondrial renewal and selective clearance. Under high‐energy demand or stress conditions, mitochondrial networks are rapidly remodeled to adapt to metabolic changes [114]. In microglia, maintaining the balance between fusion and fission is more critical than the magnitude of either process individually. Following stroke, impaired mitochondrial dynamics in microglia can exacerbate oxidative stress and metabolic disturbances, contributing to secondary brain injury [115, 116].

Mitochondrial fission is primarily regulated by dynamin‐related protein 1 (Drp1), which is predominantly a cytoplasmic protein. Outer mitochondrial membrane proteins, including mitochondrial fission 1 (Fis1) and mitochondrial fission factor (Mff), act as Drp1 anchors [117, 118]. Excessive fission disrupts mitochondrial architecture, upregulating mtROS, depleting ATP, damaging ΔΨm, and promoting cytochrome C release and apoptosis [119]. Such fission‐dominant remodeling is frequently observed during the acute inflammatory phase after stroke and is closely associated with pro‐inflammatory microglial activation. Recent studies have reported that E2F1/CDK5 and JAK2/STAT3 act as upstream regulators of Drp1 in microglia during cerebral I/R injury [120, 121]. After ICH, mitochondrial ATP synthase coupler 6 (ATP5J) upregulates Drp1 and Fis1, worsening mitochondrial fragmentation and microglial activation [122].

Conversely, fusion is mediated by mitofusin1 (Mfn1), mitofusin2 (Mfn2), and optic atrophy 1 (OPA1) [118, 123]. Fusion‐dominant remodeling is generally associated with metabolic recovery and resolution of inflammation during the reparative phase. In studies using neuroinflammatory drugs for ICH, pterostilbene was found to reverse OPA1 downregulation, promote fusion, restore normal mitochondrial morphology, reduce fragmentation and ROS production in microglia, thereby conferring neuroprotection [124].

Overall, impaired mitochondrial dynamics are closely linked to microglial activation and neuroinflammation [125, 126]. Therapeutic strategies targeting mitochondrial dynamics offer a promising avenue to restore mitochondrial function, minimize cellular damage, and promote recovery. Notably, traditional Chinese medicinal compounds have shown significant potential in modulating mitochondrial dynamics and mitigating disease progression.

### 7.4 Protein‐Level Mitochondrial Stress Responses

Beyond these organelle‐level quality control mechanisms, protein‐level stress responses and inter‐organelle communication also constitute buffering systems. These mechanisms are rapidly activated after stroke but cannot effectively prevent the progression of mitochondrial dysfunction and suppress the amplifying effects of neuroinflammation.

Mitochondrial stress at the protein homeostasis level activates the mitochondrial unfolded protein response (UPR^mt^). This adaptive transcriptional program maintains mitochondrial protein homeostasis by upregulating molecular chaperones, mitochondrial proteases, and pathways enhancing metabolic resilience [127]. Although definitive evidence supporting UPR^mt^ activation in microglia after stroke remains limited, its neuroprotective effects have been validated in several experimental models of ischemic stroke [128, 129, 130]. Recent transcriptomic analyses also identified and validated CLEC4D as a highly sensitive biomarker of the mitochondrial unfolded protein response in patients with ischemic stroke [131], suggesting potential translational significance for UPR^mt^‐related pathways.

## 8 Targeting Microglial Mitochondria Dysfunction After Stroke

Mitochondrial dysfunction in microglia has emerged as a promising therapeutic target for post‐stroke neuroinflammation. Key regulatory axes, including UCP2‐dependent redox control, PINK1/Parkin‐mediated mitophagy, AMPK‐driven metabolic homeostasis, STING‐centered inflammatory signaling, and microglial mitochondrial transplantation, have emerged as critical determinants of microglial fate after stroke. Therapeutic strategies targeting these mitochondrial checkpoints have shown promising efficacy in preclinical models (Table 1).

**TABLE 1:** Microglial mitochondria–targeted interventions in preclinical stroke model.

Mitochondrial target	Intervention	Disease	in vivo/in vitro	Effects
ROS, mtDNA‐Driven Inflammation	Idebenone [61]	Ischemia stroke	Microglia in vivo/Primary microglia/BV2 microglia	Inhibits mtROS production and Ox‐mtDNA accumulation, suppresses NLRP3 activation and inflammatory damage
ROS, mtDNA‐Driven Inflammation	Nordihydroguaiaretic acid [113]	Ischemia stroke	Microglia in vivo/Primary microglia	Limits mtDNA and Ox‐mtDNA synthesis, suppresses NLRP3 activation, and reduces ischemic neuroinflammation
ROS, Fusion and Fission Dynamics	Pterostilbene [124]	Intracerebral hemorrhage	Microglia in vivo/BV2 microglia	Enhances mitochondrial fusion, restores morphology, reduces superoxide, alleviating microglial inflammation
ROS	Anethole trithione [132]	Intracerebral hemorrhage	Microglia in vivo/BV2 microlia	Enhances mitochondrial ROS production, activates UCP2, suppresses neuroinflammation
ROS	Bendavia [133]	Ischemia stroke	Microglia in vivo	Inhibits ROS generation and apoptosis, restores ATP synthesis, reduces microglial activation, conferring neuroprotection
mtDNA‐Driven Inflammation, Mitophagy	rFGF21 [57]	Subarachnoid hemorrhage	Microglia in vivo	Promotes mitophagy, prevents mtDNA release, inhibits cGAS‐STING signaling, and exerts neuroprotective effects
mtDNA‐Driven Inflammation	P202, Mdivi‐1 [62]	Intracerebral hemorrhage	Microglia in vivo/BV2 microglia	Blocks AIM2–mtDNA interaction, prevents AIM2 inflammasome assembly, reduces neuroinflammation
Metabolism reprogramming	TEPP‐46 [68]	Ischemia stroke	Microglia in vivo/Primary microglia	Inhibits the interaction between PKM2 and HIF‐1α, suppresses gluconeogenesis in microglia
Metabolism reprogramming	A‐485/Lonidamine [70]	Ischemia stroke	Microglia in vivo/BV2 microglia	Lactate transferase inhibitor/HK2 inhibitor, restores mitochondrial function, attenuates neuroinflammation
Metabolism reprogramming	Vt‐MSN‐siFABP4 [80]	Intracerebral hemorrhage	Microglia in vivo	Reduces the accumulation of lipid droplets, suppresses microglial activation and neutrophil infiltration, and promotes functional recovery
Metabolism reprogramming	Axitinib [81]	Ischemia stroke	Microglia in vivo/HMC3 microglia	Increases CPT1A expression, restores microglial phagocytic function and reduces pro‐inflammatory activation
Metabolism reprogramming	Astragaloside IV [87]	Ischemia stroke	Microglia in vivo/BV2 microglia	Actives AMPK, inhibits mTOR/HIF‐1 signaling, and reduces glycolytic enzyme expression
Metabolism reprogramming	Resolvin D1 [134]	Ischemia stroke	Microglia in vivo/Primary microglia	Reprograms energy metabolism, shifts from glycolysis to OXPHOS, enhance microglial phagocytosis, reduce neutrophil accumulation and neuroinflammation
Metabolism reprogramming	Effusol [135]	Ischemia stroke	Microglia in vivo/BV2 microglia	Upregulates ATP synthase activity, targets NLRP3 to inhibit pyroptosis
Mitophagy	PINK1 NPs [136]	Ischemia stroke	Microglia in vivo/BV2 microglia	Reduces expression of mitophagy‐related proteins, enhances microglial phagocytosis, anti‐inflammatory polarization
Mitophagy	Sodium tanshinone IIA sulfonate [137]	Ischemia stroke	HAPI/BV2 microglia	Promotes autophagy‐associated proteins, suppresses pro‐inflammatory cytokines, and induces anti‐inflammatory mediators
Biogenesis	Cannabidiol [111]	Ischemia stroke	Microglia in vivo/BV2 microglia	Upregulates TFAM and CKS1B, ameliorates mitochondrial dysfunction, reduces microglial activation and neuroinflammation
Fusion and Fission Dynamics	Atractylenolide III [121]	Ischemic stroke	Microglia in vivo/Primary microglia	Inhibits Drp1 phosphorylation and mitochondrial fission, suppressing neuroinflammation
Mitochondrial transplantation	hUC‐MSC mitochondrial transplantation [138]	Ischemia stroke	Microglia in vivo	Inhibits microglial activation and provides neuroprotection
Mitochondrial transplantation	Artificial cells mitochondrial transplantation [139]	Intracerebral hemorrhage	Microglia in vivo/BV2 microglia	Suppresses pro‐inflammatory microglia, increases immunosuppressive phenotypes, restores energy metabolism, and alleviates neuroinflammation

*Note:* The table summarizes pharmacological and biological interventions modulating microglial mitochondrial function in ischemic and hemorrhagic stroke models. Listed therapies target mitochondrial pathways including ROS production, mtDNA‐Driven Inflammation, metabolisms, mitophagy, biogenesis, mitochondrial fusion/fission dynamics and mitochondrial transplantation. Their effects encompass suppression of neuroinflammation, restoration of mitochondrial homeostasis, and neuroprotection.

### 8.1 Targeting Microglial Mitochondrial Damage

UCP2 activation, including pharmacological stimulation by anethole trithione (ADT), was found to mitigate microglia‐mediated neuroinflammation [132], while microglia CBS‐derived H_2_S was shown to transiently activate UCP2 through RET‐linked superoxide signaling [140]. Other strategies targeting ROS have also shown therapeutic potential in stroke. The mitochondrial‐targeted tetrapeptide bendavia preserved mitochondrial integrity, suppressed ROS production, restored ATP levels, attenuated BBB disruption, and limited microglial activation [133]. Natural compounds, including pterostilbene (3′,5′‐dimethoxy‐resveratrol, PTE), alleviated mitochondrial oxidative stress and inflammatory responses after IS/ICH [124, 141].

Together, these findings underscore the importance of mtROS modulation in microglia, which is a direct anti‐inflammatory or antioxidant strategy and can contribute to the development of novel stroke therapies.

Restoring MQC through mitophagy represents another promising intervention strategy. Researchers proposed a strategy using PLGA‐based nanoparticles loaded with PINK1 siRNA (PINK1 NPs), which selectively targeted microglia, specifically modulated PINK1 expression, and alleviated photothrombotic ischemic damage [136]. Pharmacological interventions similarly modulated mitophagy. Sodium tanshinone IIA sulfonate (STS) upregulated PP2A in microglia, restoring mitochondrial function and downregulating apoptosis in I/R injury [137]. Recombinant fibroblast growth factor 21 (rFGF21) was shown to activate AMPK‐dependent mitophagy, improve inflammatory profiles, limit brain injury after subarachnoid hemorrhage, and prevent cytosolic mtDNA release, thereby suppressing the cGAS/STING pathway [57].

Additionally, targeting metabolic reprogramming in microglia represents a promising therapeutic avenue. Pharmacological or bioactive interventions that suppress glycolytic dependence, restore mitochondrial respiration, enhance FAO, or stabilize mitochondrial dynamics have shown robust benefits in preclinical models. For example, resolvin D1 promoted OXPHOS‐dependent energy metabolism, accelerated neutrophil clearance, suppressed neutrophil extracellular trap formation, and attenuated neuroinflammation [134]. The plant‐derived compound effusol was also reported to ameliorate ischemia‐induced mitochondrial dysfunction by enhancing ATP synthase activity and reducing ΔΨm depolarization [135]. In ICH models, ATP5J silencing suppressed mitochondrial hyperfission and aberrant mPTP opening, reduced ROS production, restored cristae morphology, and partly re‐established electron transport chain activity, thereby mitigating oxyhemoglobin‐induced mitochondrial dysfunction [122]. Astragaloside IV (AS) promoted microglial repair phenotypes by activating AMPK, inhibiting mTOR/HIF‐1 signaling, and downregulating the expression of glycolytic enzymes [87]. The PKM2 activator TEPP‐46 inhibited the nuclear translocation of PKM2 and its interaction with HIF‐1α, thereby suppressing glycolysis in microglia, promoting phagocytosis, and alleviating neuroinflammation [68].

### 8.2 Mitochondrial Transfer and Transplantation

Intercellular mitochondria transfer refers to a process in which one or more mitochondria are transferred from a donor cell to an acceptor cell. On the other hand, mitochondrial transplantation involves the isolation of mitochondria and their injection into a biological organism to improve graft survival and generate therapeutic benefits [142]. Overall, mitochondrial transfer and transplantation can directly supplement mitochondrial function and stimulate MQC pathways (Figure 4) [143, 144]. The earliest study was conducted in 1969, when researchers found that mitochondria and other organelles were transported through intercellular communication channels between oocytes [145]. Thereafter, mitochondrial transfer and transplantation were shown to preserve mitochondrial integrity and cellular viability, exhibiting therapeutic promise in neurological, cardiovascular, and respiratory diseases [146, 147].

Astrocytes establish astrocyte‐neuron/microglia transfer axes in the brain, transferring healthy mitochondria to neighboring neurons and microglia [148, 149, 150]. They also release mitochondria‐encoded bioactive peptides, such as humanin (HN), which can be internalized by recipient cells to promote a phagocytic or repair phenotype in microglia, thereby accelerating hematoma clearance after ICH [149]. Mesenchymal stem cells (MSCs) represent another major type of mitochondrial donor, delivering functional mitochondria via tunneling nanotubes (TNTs), extracellular vesicles (EVs), or other mechanisms [151, 152, 153, 154]. Mitochondria isolated from human umbilical cord MSCs (hUC‐MSCs) significantly minimized infarct volume, reduced cell death, and prevented glial activation in MCAO models [138].

Mitochondrial transfer from different organisms remains constrained by immune rejection and biocompatibility concerns, which substantially limit the translational applicability of mitochondrial transfer. To overcome this issue, Zhou et al. proposed a novel strategy using intravenous magnetic‐responsive artificial cells (ACs). In an external magnetic field, ACs rapidly accumulate in the brain and gradually degrade to release mitochondria. In a mouse model of ICH, these mitochondria were internalized by microglia, increasing the proportion of immunosuppressive microglia while reducing the abundance of pro‐inflammatory microglia and neuroinflammation. Thus, brain edema was attenuated, and functional recovery was enhanced [139]. Nevertheless, recent quantitative analyses have shown that most exogenous mitochondria remain sequestered in the endosomal‐lysosomal system, with fewer than 10% successfully escaping into the cytosol and integrating into the host mitochondrial network. This finding suggests that inefficient endosomal escape represents a critical bottleneck limiting therapeutic efficacy [155].

From a translational perspective, mitochondrial transplantation has recently entered an early‐stage clinical trial. In patients with acute IS undergoing mechanical thrombectomy, viable autologous mitochondria were rapidly isolated and delivered intra‐arterially to the affected vascular territory, demonstrating preliminary safety and feasibility [156]. Tc‐99 m–labeled mitochondrial tracing further provides a methodological approach for monitoring the in vivo biodistribution and homing efficiency of freshly isolated mitochondria [157].

To minimize immunogenicity, available clinical approaches primarily rely on autologous mitochondrial sources. However, key parameters, including optimal dosing, timing of administration, the feasibility of repeated dose delivery, and the potential of allogeneic transplantation, remain to be systematically defined.

Future efforts should focus on improving donor mitochondrial quality, developing targeted delivery systems that can enhance endosomal escape, and rigorously evaluating immunogenicity and mitochondrial‐nuclear compatibility. Standardized manufacturing protocols and robust translational pipelines are essential for advancing mitochondrial therapies toward reliable and widespread clinical application.

## 9 Discussion and Perspectives

Drawing upon existing evidence, we propose that microglial mitochondrial dysfunction represents a conceptual “storm center” within the complex pathogenesis of stroke. This framework provides a conceptual basis for understanding how mitochondrial dysfunction shapes the evolution of post‐stroke neuroinflammation. Importantly, the contribution of microglial mitochondria is likely context‐dependent and may vary across stroke subtypes, brain regions, and temporal stages.

Despite significant progress, several critical gaps remain in translating microglial mitochondrial research into the clinical treatment of stroke. First, microglial mitochondria‐targeted therapies for stroke have not been investigated in clinical trials, and clinically applicable biomarkers or imaging tools to monitor mitochondrial function in microglia are lacking. Current evidence is largely derived from therapeutic clinical trials and emerging imaging studies, providing only indirect and fragmented insights, and overall clinical validation remains limited. Therapeutic clinical trials and emerging imaging approaches provide only fragmented insight into mitochondrial states in microglia in stroke (Tables 2 and 3).

**TABLE 2:** Mitochondria‐related therapeutic clinical evidence in stroke.

Evidence type	Disease and intervention	Participants or studies (*N*)	Study design and endpoints	Outcomes	References
Phase II RCT	IS; Cyclosporine A (mPTP inhibitor)	127 patients	Adjunct to IV thrombolysis; infarct volume assessed at 30 days	No overall benefit; reduced infarct size in reperfused subgroup	[158]
Double‐blind RCT	IS; Coenzyme Q10 (mitochondrial antioxidant)	50 patients	Treatment initiated ≤ 24 h; outcomes assessed at 30 days	Reduced oxidative stress markers; possible neurological improvement	[159]
Pilot RCT	IS; Melatonin (mitochondrial antioxidant)	Pilot cohort	Early treatment; follow‐up at 1–3 months	Improved short‐term neurological recovery signals	[160]
Systematic review/meta‐analysis	IS; Edaravone (ROS scavenger)	19 studies	Functional outcomes (e.g., mRS) pooled	Suggested benefit; further high‐quality trials required	[161]
Systematic review/meta‐analysis	ICH; Edaravone	38 RCTs; 3454 patients	Mortality and functional outcomes analyzed	No mortality benefit; insufficient long‐term outcome data	[162]
Meta‐analysis	IS; Edaravone + Dexborneol	Multiple studies	90‐day functional outcomes pooled	Combination associated with improved recovery	[163]

*Note:* This table compiles clinical therapeutic evidence targeting mitochondrial dysfunction and oxidative stress in ischemic and hemorrhagic stroke, including randomized controlled trials, systematic reviews, and meta‐analyses. Although no therapies directly targeting microglial mitochondrial pathways have yet entered routine clinical practice, these studies provide translational benchmarks and support mitochondrial mechanisms as potential therapeutic targets.

**TABLE 3:** Neuroinflammation and mitochondrial imaging studies.

Imaging modality	Study population	Participants (*N*) and imaging protocol	References
TSPO‐PET (11C‐PK11195)	Ischemic stroke patients	6; PET performed 3–150 days post‐stroke	[164]
TSPO‐PET + DTI	Ischemic stroke patients	18; imaging within ≤ 2 weeks and repeated at 6 months	[165]
TSPO‐PET (11C‐PBR28)	Chronic ischemic stroke patients + healthy controls	8 patients +16 controls; scans acquired 1–3 years post‐stroke	[166]
18F‐BCPP‐EF PET (mitochondrial complex‐I tracer)	Healthy volunteers	12; dynamic PET acquisitions for tracer characterization	[167]
Tc‐99 m mitochondrial radiotracer	Isolated mitochondria (ex vivo preparation)	NA; First‐in‐field feasibility protocol	[157]

*Note:* This table summarizes in vivo imaging approaches for assessing neuroinflammation and mitochondrial function in stroke cohorts. Modalities include TSPO‐PET for microglial activation, mitochondrial complex‐I PET tracers, and exploratory mitochondrial radiolabeling techniques. These imaging tools enable longitudinal monitoring of inflammatory activity and mitochondrial bioenergetics across acute and chronic stages of stroke.

Second, the optimal therapeutic time window for mitigating secondary brain injury remains unknown, potentially due to phase‐like transitions in microglial state and mitochondrial metabolism across different time points and brain regions. Most existing studies focus predominantly on the acute phase. Future investigations should adopt phase‐resolved and region‐specific designs to identify both effective therapeutic windows and potential risk periods for mitochondrial modulation.

Third, our current understanding heavily relies on rodent models, which cannot fully capture the complexity, heterogeneity, and multifactorial nature of human stroke. Future studies should leverage publicly available stroke‐related single‐cell immune datasets (Table 4) and apply integrative machine learning approaches to identify key mitochondria‐related genes and pathways. Additionally, a novel three‐dimensional human brain tissue model has recently been developed, which provides a platform for studying immune cell interactions in a microenvironment closer to that of humans and allows for drug screening [176].

**TABLE 4:** Human brain and blood multi‐omics profiling studies in stroke.

Data type	Disease and specimens	Key design features	References
Single‐cell RNA‐seq	ICH; perihematomal edema (PHE) brain tissue immune cells (microglia/myeloid enriched)	9 patients; Surgical tissue; 0–48 h after onset	[38]
Single‐cell RNA‐seq	ICH; hematoma drainage fluid + peripheral blood leukocytes	1 patient; Longitudinal serial sampling	[168]
Bulk transcriptomics (microarray)	ICH autopsy brain; perihematoma vs. contralateral gray/white matter	4 autopsy cases; acute stage	[169]
Spatial transcriptomics (Xenium)	Human carotid atherosclerotic plaques (major upstream substrate for ischemic stroke)	Multiple surgical specimens; single‐cell spatial mapping	[170]
Blood transcriptomics	AIS; whole blood	= 70 patients; ≤ 24 h after onset vs. controls	[171]
Plasma proteomics	AIS; plasma	Paired pre/post rt‐PA (1 h) within individuals	[172]
Serum proteomics	AIS vs. ICH; serum	< 24 h after onset; large discovery + validation cohorts	[173]
Serum proteomics	AIS; serum	Outcome‐stratified cohort; 3‐month recovery	[174]
Targeted metabolomics	IS; Population plasma cohort	*n* = 2378; prospective long‐term follow‐up	[175]

*Note:* This table summarizes patient‐derived multi‐omics datasets generated from human brain tissue and peripheral blood collected in clinical stroke cohorts. These studies provide direct molecular and cellular evidence from human disease, enabling translational characterization of microglial heterogeneity, neuroinflammation, and mitochondrial‐related metabolic dysfunction.

Fourth, interventions delivery shows limited efficiency in crossing the BBB or specifically targeting microglia. Effective therapies must cross the blood–brain barrier, selectively target microglia, and localize to mitochondria, yet many current approaches suffer from poor brain penetration, low specificity, and suboptimal pharmacokinetics. The development of mitochondria‐targeted delivery systems, such as RNP–MITO‐Porter nanocarriers and mitochondria‐specific base editors, provides technical advancement [177, 178]. However, their effective delivery and specificity for microglia in the context of stroke necessitate further validation.

Translation of preclinical discoveries into clinical practice necessitates standardized phenotyping pipelines in human post‐stroke samples, combining metabolomics, spatially resolved mitochondrial activity assays, and longitudinal in vivo imaging of microglia–neuron–glia interactions. Finally, these technological and conceptual advances may enable precision modulation of microglial mitochondrial pathways, supporting novel therapeutic strategies to restore mitochondrial homeostasis and attenuate the neuroinflammatory cascade underlying secondary brain injury.

## Author Contributions

**Ruchong Fan:** writing – original draft, conceptualization, investigation. **Chuan Wang:** conceptualization, visualization. **Zi Lin:** investigation, visualization. **Shiling Chen:** conceptualization, investigation. **Chao Pan:** writing – review and editing. **Hao Nie:** writing – review and editing. **Chuan Qin:** conceptualization, Investigation. **Xuan Wu:** writing – review and editing. **Zhouping Tang:** conceptualization, funding acquisition.

## Funding

This work was supported by the National Natural Science Foundation of China (Grants: 92148206) and the Major Program (JD) of Hubei Province (Grants: 2023BAA005).

## Ethics Statement

The authors have nothing to report.

## Conflicts of Interest

The authors declare no conflicts of interest.

## Data Availability

Data sharing not applicable to this article as no datasets were generated or analysed during the current study.
